# WIC staff and healthcare professional perceptions of an EHR intervention to facilitate referrals to and improve communication and coordination with WIC: A qualitative study

**DOI:** 10.1017/cts.2024.488

**Published:** 2024-02-26

**Authors:** Abigail McCall, Ashley E. Strahley, Katy W. Martin-Fernandez, Kristina H. Lewis, Angelina Pack, Beatriz Ospino-Sanchez, Ivy Greene, Gabriela de la Vega, Alysha J. Taxter, Sally G. Eagleton, Kimberly G. Montez

**Affiliations:** 1 Section on General Academic Pediatrics, Department of Pediatrics, Wake Forest University School of Medicine, Winston-Salem, NC, USA; 2 Department of Social Sciences and Health Policy, Division of Public Health Sciences, Wake Forest University School of Medicine, Winston-Salem, NC, USA; 3 Department of Surgery, Wake Forest University School of Medicine, Winston-Salem, NC, USA; 4 Department of Epidemiology and Prevention, Division of Public Health Sciences, Wake Forest University School of Medicine, Winston-Salem, NC, USA; 5 Department of Implementation Science, Division of Public Health Sciences, Wake Forest University School of Medicine, Winston-Salem, NC, USA; 6 Division of Rheumatology, Department of Pediatrics, Nationwide Children’s Hospital, Columbus, OH, USA; 7 Division of Clinical Informatics, Department of Pediatrics, Nationwide Children’s Hospital, Columbus, OH, USA; 8 Clinical and Translational Science Institute, Wake Forest University School of Medicine, Winston-Salem, NC, USA

**Keywords:** Electronic health record, food insecurity, primary care, pediatrics, WIC

## Abstract

**Objectives:**

Participation in the Special Supplemental Nutrition Program for Women, Infants, and Children (WIC) has numerous benefits, yet many eligible children remain unenrolled. This qualitative study sought to explore perceptions of a novel electronic health record (EHR) intervention to facilitate referrals to WIC and improve communication/coordination between WIC staff and healthcare professionals.

**Methods:**

WIC staff in three counties were provided EHR access and recruited to participate. An automated, EHR-embedded WIC participation screening and referral tool was implemented within 8 healthcare clinics; healthcare professionals within these clinics were eligible to participate. The interview guide was developed using the Consolidated Framework for Implementation Research to elicit perceptions of this novel EHR-based intervention. Semi-structured interviews were conducted via telephone. Interviews were recorded, transcribed, coded, and analyzed using thematic analysis.

**Results:**

Twenty semi-structured interviews were conducted with eight WIC staff, seven pediatricians, four medical assistants, and one registered nurse. Most participants self-identified as female (95%) and White (55%). We identified four primary themes: (1) healthcare professionals had a positive view of WIC but communication and coordination between WIC and healthcare professionals was limited prior to WIC having EHR access; (2) healthcare professionals favored WIC screening using the EHR but workflow challenges existed; (3) EHR connections between WIC and the healthcare system can streamline referrals to and enrollment in WIC; and (4) WIC staff and healthcare professionals recommended that WIC have EHR access.

**Conclusions:**

A novel EHR-based intervention has potential to facilitate healthcare referrals to WIC and improve communication/coordination between WIC and healthcare systems.

## Introduction

Adequate nutrition during the first 1,000 days of a child’s life, the period of the most rapid neuronal proliferation, is essential for healthy growth and development [1]. The Special Supplemental Nutrition Program for Women, Infants, and Children (WIC) is a federally funded program that provides nutritional and breastfeeding support and counseling to low-income, pregnant and lactating individuals, and infants and children up to 5 years of age [2]. WIC participation improves dietary quality and reduces food insecurity, and has been associated with reduced risk for preterm birth, low birth weight, and childhood obesity [3,4]. WIC promotes health equity by reducing food insecurity and mitigating its associated adverse outcomes, which disproportionately affect minoritized households due to structural racism, discrimination, and xenophobia [5,6].

Despite improved outcomes, only 50% of the eligible 12.5 million people participate in WIC, resulting in underutilization of benefits [7]. The WIC participation rate, defined by WIC as enrolled individuals who are using their benefits [7], has been declining since 2011 when it reached a high of over 63% [8]. A large body of prior research demonstrates the multitude of barriers to WIC participation. Challenges to participation are numerous and include misunderstandings about eligibility status, language and cultural barriers, negative WIC clinic experiences, difficulty redeeming benefits, lack of transportation to reach WIC clinics, and stigma, among other reasons [9–11]. Despite multiple waivers during the COVID-19 pandemic, such as the physical presence waiver, there were modest increases in WIC participation between March 2020 and March 2022 [12], demonstrating the need for cross-sector strategies to improve outreach and increase enrollment.

The healthcare system, particularly within primary care settings, represents a unique opportunity for healthcare professionals to connect patients to and coordinate care with WIC. Several organizations, including the American Academy of Pediatrics, recommend that pediatric healthcare professionals refer eligible patients to WIC [13–15]. Although WIC and healthcare systems care for a shared population, they have traditionally existed in information siloes, each documenting in their own secure electronic records. These siloes likely exist due to concerns about data security and patient confidentiality that create barriers to data sharing. Because of these siloes, communication and coordination between these entities have historically required multiple phone calls or faxes, and reliance upon patients as intermediaries. This scenario can lead to confusion and care delays, and potentially reduce the effectiveness of nutritional counseling. Despite the potential for healthcare system connections with WIC, little research exists evaluating healthcare system-based connections to WIC [16–19], and even less on interventions to improve WIC enrollment [20].

To streamline the healthcare referral process to WIC and promote improved communication and care coordination between WIC staff and healthcare professionals, our healthcare system recently piloted an innovative EHR-based intervention. The purpose of this study is to qualitatively explore perceptions of this novel intervention among WIC staff and healthcare professionals.

## Methods

### Study setting

This qualitative study was part of a prospective investigation in which our team implemented an innovative EHR-based WIC screening and referral intervention to improve communication and care coordination at eight healthcare clinics within the Atrium Health Wake Forest Baptist system, a large healthcare system serving a racially and ethnically diverse patient population within the western portion of North Carolina that uses a fully integrated EHR system (Epic©). Table 1 outlines the patient demographics of each of these healthcare clinics. Race, ethnicity, and preferred language for healthcare use were self-reported. To estimate socioeconomic status, we utilized the “economically disadvantaged student” (EDS) data from the North Carolina Department of Public Instruction. EDS is defined as “any student identified by a Public School Unit meeting the criteria of Directly Certified, Categorically Eligible, or a method consistent with the state or federal guidance for financial assistance regardless of participation or eligibility in the National School Lunch Program [21].” Higher percentages of EDS indicate that the school has a higher proportion of students with socioeconomic disadvantage. For each healthcare clinic, we used the school closest to the clinic to determine the corresponding EDS data for the most recently available school year [22].

**Table 1.** Patient characteristics from participating healthcare clinics

Clinic	1	2	3	4	5	6	7	8
***n***	14,968	7,448	13,690	12,881	3,657	4,044	5,802	1,099
**Race**								
AI/AN	0.3%	0.1%	0.4%	0.6%	0.6%	0.4%	0.3%	0.2%
Asian	1%	0.3%	2%	3%	3%	1%	2%	2%
Black	28%	63%	19%	16%	13%	16%	43%	39%
NH/PI	0.3%	0.2%	0.2%	0.2%	0.4%	0%	0%	0.2%
Other	54%	28%	25%	6%	18%	28%	20%	15%
White	16%	8%	53%	74%	65%	55%	37%	42%
Unknown	0.4%	0.4%	0.4%	0.2%	1%	0.6%	0.7%	0.6%
**Ethnicity**								
Hispanic	51%	24%	20%	6%	17%	24%	13%	8%
Non-Hispanic	48%	75%	78%	93%	81%	75%	86%	88%
Other	1%	1%	2%	1%	2%	1%	1%	4%
**Language**								
English	59%	84%	90%	98%	93%	86%	85%	95%
Spanish	39%	15%	9%	1%	6%	13%	13%	2%
Other	2%	1%	1%	1%	1%	1%	2%	3%
**EDS**	16%	≥90%	≥90%	≥90%	≥90%	42%	≥90%	15%

AI/AN = American Indian/Alaska Native; EDS = economically disadvantaged student; NH/PI = Native Hawaiian/Pacific Islander.

Care coordination is defined as “deliberately organizing patient care activities and sharing information among participants concerned with patient’s care to achieve safer and more effective care [23].” Under an existing memorandum of understanding (MOU) with the healthcare system, WIC staff in three counties were granted secure, limited online access to the healthcare system’s EHR in 2020 through Epic CommunityLink©, which is a provider portal designed to improve communication and enhance patient care [24,25]. This access allowed users to view WIC clients’ medical charts, send and receive secure messages with healthcare teams, and receive WIC referrals from the healthcare system. WIC staff were provided training sessions by healthcare system staff on how to utilize the online EHR.

Increasingly, healthcare systems are utilizing nudges, which are subtle modifications to the design of the environment or information framing that can influence healthcare professionals’ behavior. These nudges, such as default options to increase generic prescribing, can be used to improve health care delivery and patient outcomes by leveraging the EHR to implement scalable, innovative interventions [26]. Our intervention utilized a nudge. For children under five years of age with Medicaid or no insurance, an EHR-embedded tool was implemented during well-child checks at the time of the visit. At seven clinics within the healthcare system, an automated alert prompted medical assistants (MAs) one time during the rooming process to screen for WIC enrollment and assess interest in referral if not enrolled (October 2021). Research staff provided training sessions on how to effectively screen and refer using the tool, as well as how to counsel families. At one academic clinic within the healthcare system, pediatric residents, advanced practice providers, and faculty were prompted to screen via an EHR-embedded tool within the progress note template, which has been previously described [20]. For consenting families, automated referrals were sent directly to WIC’s EHR inbox to begin enrollment. Family consent for referral was documented in the EHR.

This study was conducted in collaboration with three local Departments of Public Health (DPH) WIC program leadership as part of a long-standing MOU. For example, the DPH and healthcare system have long shared client and patient populations as the county’s safety net primary care clinic was previously operated by the DPH but over 20 years ago the healthcare system assumed operation of the clinic, necessitating the creation of the MOU. This MOU facilitated data sharing between the DPH and healthcare system. Additionally, many healthcare system physicians continue to staff multiple other healthcare clinics within the DPH.

### Framework, participants, and data collection

Individual semi-structured interviews were deemed the most appropriate method of data collection due to the individual nature of screening, referral, enrollment, and patient communication experiences, as well as the ease and ability to schedule individual interviews. Given the multilevel nature of the screening, referral, and communication processes between WIC staff and healthcare professionals (physicians/advanced practice providers treating pediatric patients < 5 years of age, MAs), and nurses (both registered nurses [RNs] and licensed practical nurses [LPNs]), as well as the contextual factors that facilitate or hinder implementation processes, we sought to capture organizational characteristics impacting intervention implementation. Therefore, the Consolidated Framework for Implementation Research (CFIR) was applied to elicit perceptions of the intervention via semi-structured interviews [27]. The CFIR framework includes 39 factors across five domains of intervention characteristics (i.e., innovation, outer setting, inner setting, implementation process, and individuals). These domains influence intervention implementation at the systems level and have been applied in qualitative research, including among WIC participants [11,28]. Given the formative phase of this project, the semi-structured interview guide was developed to incorporate CFIR domains to guide future endeavors.

Through a detailed review of the literature [11,17], consultation with outside experts, and input from WIC staff, we developed an interview guide to elicit WIC staffs’ and healthcare professionals’ perceptions of the EHR-based intervention. Domains of inquiry were mapped to CFIR constructs. Questions explored WIC staffs’ experiences in communicating with clients and healthcare professionals before (CFIR: outer setting) and after the intervention (CFIR: innovation), how the intervention affected client referrals, enrollment, and recertification as well as service provision (CFIR: innovation), feedback about the intervention (CFIR: implementation process), and whether the EHR-based intervention was recommended for other WIC programs (CFIR: implementation process). For healthcare professionals, questions explored the knowledge and perception of the WIC program (CFIR: outer setting), experiences in communicating and coordinating care with WIC staff and sending referrals before (CFIR: inner setting) and after the intervention (CFIR: innovation), feedback about screening for WIC enrollment and sending referrals within clinical healthcare settings (CFIR: implementation process), how the intervention affected WIC referrals and service provision (CFIR: innovation), and whether the EHR-based intervention was recommended for other healthcare systems (CFIR: implantation process). Self-reported participant demographics were collected at the time of the interview (CFIR: individuals). The semi-structured interview guide was pilot-tested for face validity with representative participants and modified iteratively.

The sampling strategy was purposive based on inclusion criteria, utilized an opt-in approach, and ensured participant consent. All WIC staff from three participating counties and healthcare professionals from eight participating clinics (healthcare professionals) were included in the study. Recruitment occurred via an email invitation to participate in a semi-structured interview via telephone. Recruitment flyers with a quick response code were also posted in MA/RN workspaces in participating clinics. Twenty-one WIC staff from three counties were eligible. Forty-one pediatricians, 35 RN/LPNs, and 34 MAs were eligible. These health professionals were chosen based on their various roles in engaging patients with WIC. For example, at seven clinics in the health system, MAs screen for participation, refer to WIC, and counsel about WIC services; RN/LPNs assist with communication between WIC staff and healthcare professionals, such as answering/making phone calls from/to WIC, responding to inbox messages, and sending/receiving faxes; pediatricians counsel families about WIC, send referrals, and communicate with WIC about patients, and in one clinic in the healthcare system, they screen for participation and refer to WIC. Interested individuals opted-in to the study by contacting study personnel directly via email to schedule an interview. Interviews were scheduled at the participant’s convenience.

Two researchers (A.P. and B.S.), who were trained by our institution’s professional qualitative research team (QPRO) on qualitative interview techniques, conducted telephone-based interviews in English using the interview guide. Informed consent was obtained by telephone. Given research staffing, WIC staff were interviewed between September and November 2022, and healthcare professionals were interviewed between December 2022 and February 2023. Interviews were conducted until data saturation was reached, defined as the degree to which new data repeated what was expressed in previous data [29]. Interviews lasted approximately 20 minutes (range 15–24 minutes). Each participant was compensated with a $25 gift card, provided by mail after completion of the interview. All interviews were audio-recorded, transcribed, de-identified, and verified against the audio recording.

### Research team and reflexivity

Our multi-disciplinary research team included clinicians, informaticists, students, and researchers with expertise in health disparities, implementation science, and qualitative methodologies. The interviews were conducted by authors A.P. and B.S., research staff members who were naïve to the research participants. Interview participant identification data were blinded to all other members of the research team. However, it is possible that two members of the research team (K.H.L. and K.G.M.) knew or had previously worked with some of the WIC staff and pediatricians interviewed, but this could not be ascertained due to blinding of participant identification. In order to establish the WIC screening and referral program, these two researchers worked closely with three WIC staff members at two local WIC offices who were possibly interviewed. The data collectors and analysts were trained in qualitative research methodology by our institution’s qualitative research shared resource, QPRO.

### Data analysis

Raw narrative data from the interview transcripts were entered into Atlas.ti (version 23 software, Scientific Software Development GmbH, Berlin, Germany) for data analysis. A coding scheme and dictionary were developed from the first five interviews. We used a combined inductive-deductive thematic analysis approach to code interviews, a technique that systematically describes qualitative data. Codes were derived deductively from the research questions and the interview guide and were also created inductively as the code emerged from the data. Three researchers (A.M., A.S., K.M.F.), including one from QPRO, coded each transcript independently and assigned codes to specific responses in each transcript based on the coding scheme. Discrepancies in coding were discussed among the three coders and resolved iteratively. The codebook was adjusted, as needed, based on discussions of code meanings and application. Segments of text were reviewed by code or groups of codes and summarized. Summaries were synthesized into themes using the principles of thematic analysis [30]. Themes were mapped to CFIR constructs. The Wake Forest University School of Medicine Institutional Review Board approved this study.

## Results

Twenty semi-structured interviews were conducted with eight WIC staff from two counties, seven pediatricians, four MAs, and one nurse. Most participants self-identified as female (95%) and White (55%); 30% self-identified as Hispanic and 35% spoke Spanish (Table 2). We identified four primary themes, which were mapped to CFIR constructs: (1) healthcare professionals had a positive view of WIC (CFIR: inner setting) but communication and coordination between WIC and healthcare professionals were limited prior to WIC having EHR access (CFIR: outer setting); (2) healthcare professionals favored WIC screening using the EHR but workflow challenges existed (CFIR: implementation process); (3) EHR connections between WIC and the healthcare system can streamline referrals to and enrollment in WIC (CFIR: innovation); and (4) WIC staff and healthcare professionals recommended that WIC have EHR access (CFIR: implementation process). Within these themes, we identified several subthemes that are supported by representative quotes (Table 3) and mapped to CFIR constructs.

**Table 2.** Participant demographics

(*N* = 20)	*N* (%)
**Gender**	
Female	19 (95%)
Male	1 (5%)
**Race**	
Black	1 (5%)
Asian-Indian	1 (5%)
White	17 (85%)
Other	1 (5%)
**Ethnicity**	
Hispanic	6 (30%)
Non-Hispanic	14 (70%)
**Language other than English spoken**	
None	12 (60%)
Spanish	7 (35%)
Other	1 (5%)
**Educational Level**	
Some College	5 (25%)
Associate’s Degree	1 (5%)
Bachelor’s Degree	3 (15%)
Master’s Degree	4 (20%)
Professional Degree	7 (35%)
**Provider Role**	
Physician	7 (35%)
MA or RN/LPN	5 (25%)
WIC staff	8 (40%)
**Years of Clinical Experience**	
0–4	7 (35%)
5–9	3 (15%)
10–19	4 (20%)
20+	6 (30%)

MA = Medical Assistant; RN = Registered Nurse; WIC = Special Supplemental Nutrition Program for Women, Infants, and Children.

**Table 3.** Themes, subthemes, and representative quotes

Theme	Subtheme	Representative Quote
**Healthcare professionals had a positive view of WIC (CFIR: inner setting) but communication and coordination between WIC and healthcare professionals was limited prior to WIC having EHR access (CFIR: outer setting)**	*Healthcare professionals understood WIC benefits and voiced their helpfulness (CFIR: inner setting)*	“Overall, it’s an excellent program. It offers nutritional advice to families who otherwise do not have access to such advice. It provides formula to the moms who are not breastfeeding and lactation consultant support for breastfeeding mothers, so I think very highly of the program.” (Pediatrician 02)
		“I think it’s great to have this resource available to parents for those who need it, especially pregnant, women, breastfeeding women, and those who can’t breastfeed.” (MA 03)
		“I think it’s great because it helps people afford things that they can’t normally afford.” (MA 02)
		“It doesn’t change my opinion. It kind of makes me feel good that they do know how to reach out and get resources and help from funding and government assistance in the community.” (MA 01)
	*Communication and coordination were limited between WIC and healthcare prior to the intervention (CFIR: outer setting)*	“The difficulty was the consistency of having to leave multiple messages of what we needed, and then have the prescription sent incorrectly, and then have to reach back out. Unfortunately, this communication delayed a corrected prescription, which meant it delayed services that we could provide to the customer.” (WIC staff 07)
		“Well, it often depends on the availability of that provider. It could be maybe right away, if we were lucky. It could be hours, or it could be days to get in touch with the provider.” (WIC staff 05)
		“I think the only barrier is timing of the phone calls. The way phone calls work in our clinic is that, say if a nutritionist wanted to talk to me, they would call in, and get ahold of the triage nurse. The triage nurse would then route the message to me, and then depending on what my day was looking like, I would call them back at a later time, and sometimes they’re not available. So, sometimes we would leave voice messages for one another.” (Pediatrician 05)
		“It was tough. [laughs] Obviously, everyone is very busy so finding a time when everyone is free to be able to chat on the phone was a challenge. It was harder to get in touch with the exact person that you needed to.” (Pediatrician 06)
**Healthcare professionals favored WIC screening using the EHR but workflow challenges existed (CFIR: implementation process)**		“For every zero- to five-year-old visit, I ask if they are currently receiving WIC, and if they’re not, we follow up with the question, “Would you like to be referred to WIC?”…If I click yes, that automatically sends the referral to WIC.” (Pediatrician 01)
		“Time is sometimes a barrier, and it is part of our standardized workflow. I think it’s less missed because of that, but sometimes if it’s a quick visit or if the patient was late, I could see how that might be a barrier.” (Pediatrician 04)
		“It’s not easy because if we click on a patient to look at their chart before we go in and room that patient, the question comes up, and if we disregard it, we can’t get it back. We have to go and find the flowsheet in the chart to get back to answer the question.” (MA 05)
		“A lot of people don’t feel comfortable with answering questions like that if they are getting assistance, so I put it into my own words to make them feel comfortable with answering the question.” (MA 03)
**EHR connections between WIC and the healthcare system can streamline referrals to and enrollment in WIC (CFIR: innovation)**	*Prior to the EHR connection, sending referrals to WIC was burdensome on healthcare professionals (CFIR: outer setting) but improved after the intervention (CFIR: innovation)*	“We have to receive [referrals] from a fax, or [the provider] would have to call individually to the program, or they would provide a prescription to the customer so that they would bring it to us.” (WIC staff 07)
		“We would usually receive it— either just a patient would contact us—or another program that a client was interacting with may contact us; but we weren’t necessarily communicating directly with a provider’s office.” (WIC staff 02)
		“I think from our end the biggest barrier was the way these faxes were getting sent out…we’d have to fill it, make sure it gets sent, so there were many layers to sending this form over to WIC.” (Pediatrician 05)
		“I just think it’s made referrals a lot easier because I ask the question as part of the visit and the referral happens at the same moment…as the visit with the family. It’s a fast referral…It’s not getting lost. It reduces the burden on me for completing paperwork and talking to staff. It also makes it easier for families because that referral is happening automatically.” (Pediatrician 01)
		“This [the EHR referral workflow] is just so streamlined…It just takes a lot of that extraneous, extra work out of our day that it’s very appreciated.” (Pediatrician 04)
	*Prior to the EHR connection, WIC staff felt enrollment and recertification were not difficult (CFIR: outer setting), but access to the EHR enabled obtaining required information that made the process more efficient and accurate (CFIR: innovation), while healthcare professionals felt there were barriers (CFIR: outer setting)*	“Not really difficult in respect to being enrolled, but more kinda difficult in respect to the nutrition assessment [for recertification]. Specifically with the anthropometrics, it was very difficult to get something that was updated so we weren’t really sure how adequate or how appropriate this child was growing because we didn’t have anything to refer back to, just mother’s word.” (WIC staff 01)
		“Prior to the pandemic there would be more difficulty as far as [clients] having to come into the office so transportation and getting them off working and getting kids off of daycare and things like that presented barriers [that] don’t really exist currently with [COVID] waivers.” (WIC staff 02)
		“No, I did not hear any barriers from the client to enroll in the program. For the referral, we would just call them.” (WIC staff 03)
		“Having their measurements for the recertification is helpful because we are doing some of these assessments over the phone.” (WIC staff 04)
		“I think there was a lot of concern…for families who are undocumented. I think there was concern that whether using that benefit would have negative effects later on.” (Pediatrician 01)
		“Prior to COVID, they had to go in person for visits, and that was very difficult because the families are working, and then they have to go in person for visits. It can be challenging to get that all to work out. Now they can be doing more virtually, and I think that’s been helpful for a lot of families. I think we’ve seen better engagement with WIC since that happened.” (Pediatrician 01)
		“…the only barrier I can see is the emotional side of it. Some people may be embarrassed, shameful. Their pride will not accept it.” (MA 01)
**WIC staff and healthcare professionals recommended that WIC have EHR access (CFIR: implementation process)**	*WIC staff and healthcare professionals expressed that access to the EHR may improve communication and coordination (CFIR: innovation)*	“Because of COVID we don’t have measurements for the kids since they were born, literally, so sometimes having the real data because the parent can’t recall exact measurements is very beneficial because we can assess growth data.” (WIC staff 03)
		“Oh, a hundred percent. I mean, this [EHR access] should be a mandate. This is amazing. I’m just so grateful.” (WIC staff 05)
		“I love the way that the approach is a wholeness approach versus individual approaches. It helps us as nutritionists to feel a little bit more with back up from the medical community – feeling supported by the medical community. I feel that we are more efficient, or the message gets more accepted because we work together in a way with the medical community because of the support. I think that has really helped.” (WIC staff 05)
		“I think it really helps with the referral process…I think there is less likelihood of it to get dropped, and hopefully there is, that means that higher percentage of referrals that are made actually get followed through with and families are able to access the service.” (Pediatrician 04)
		“Oh 100%, I think it’s been very helpful, and I think it also presents the opportunity for two-way communication as well in the future, which I think will be really beneficial.” (Pediatrician 01)
		“It may be helpful, but I don’t think it should be a requirement.” (MA 02)
	*Direct messaging between WIC and healthcare professionals via the EHR could potentially improve communication (CFIR: innovation), but many were unaware of the function (CFIR: individuals)*	“I haven’t really been dealing with a lot of prescriptions recently, so I haven’t been needing to send the provider any messages. But I had in the past sent them some messages…in respect to a prescription that we had received. Unfortunately, they never got back to me…so I had to call them.” (WIC staff 01)
		“I would say [I communicate with WIC] less because, mainly because the referral process is so streamlined now, I probably would have been faxing more from a referral standpoint before that was available.” (Pediatrician 04)
		“I don’t send them [messages] because I don’t know how. I wasn’t aware that I could send communication, messages through [the EHR].” (Pediatrician 07)
		“I just haven’t any questions, I haven’t had a need to reach out.” (Pediatrician 06)
		“Since the link between the two, we haven’t really gotten any phone calls anymore saying that they need an order sent over.” (MA 03)

COVID = Coronavirus disease 2019; MA = Medical Assistant; RN = Registered Nurse; WIC = Special Supplemental Nutrition Program for Women, Infants, and Children.

### Theme 1: healthcare professionals had a positive view of WIC (CFIR: inner setting) but communication and coordination between WIC and healthcare professionals were limited prior to WIC having EHR access (CFIR: outer setting)

All healthcare professionals described WIC positively, but both WIC staff and healthcare professionals described limited interactions with or difficulty coordinating care prior to the EHR intervention. We identified two subthemes within this primary theme.

#### Subtheme 1.1: healthcare professionals understood WIC benefits and voiced their helpfulness (CFIR: inner setting)

All healthcare professionals interviewed had a positive opinion of WIC, reporting that the program helped people in need and provided important nutrients to pregnant and lactating individuals and children during critical developmental times. Additionally, all healthcare professionals remarked that learning someone participates in WIC does not change their opinion about said person. Instead, they felt good knowing that a person was getting needed support. Despite overall positive attitudes about WIC, some healthcare professionals expressed the following criticisms of the WIC program: (1) delays in patient enrollment and access to WIC services; (2) difficulty enrolling and maintaining enrollment; (3) strict requirements for WIC formula prescriptions; and (4) limitations to WIC eligibility (e.g., restrictions based on lactating status, income, etc.).

#### Subtheme 1.2: communication and coordination were limited between WIC and healthcare professionals prior to the intervention (CFIR: outer setting)

##### WIC staff

WIC staff primarily communicated via phone with healthcare professionals before having EHR access, and there was often a delay in reaching them. Half of the WIC staff described communication with healthcare professionals as difficult or inefficient, and even those who did not find it difficult described it as “time-consuming.” WIC staff described back-and-forth messages with healthcare professionals and having to leave messages with a clinic nurse or receptionist rather than communicating directly with the physician or advanced practice provider. Before WIC had EHR access, the main reason that WIC staff communicated with healthcare professionals was about formula prescriptions.

##### Healthcare professionals

Healthcare professionals reported having little direct communication and coordination with WIC offices prior to WIC accessing the EHR. Healthcare professionals mostly faxed referrals to WIC without direct communication with WIC office personnel. When they did communicate with WIC, it was typically by phone, and some healthcare professionals shared that it could be difficult to get ahold of WIC staff. Prior to WIC having EHR access, healthcare professionals said that they coordinated with WIC mostly about formula prescriptions, such as requesting a specialized formula for a patient or clarifying a formula prescription (e.g., change formula because not contract approved, clarify amount of formula and/or duration of prescription, revise medical condition so that it qualifies, etc.).

### Theme 2: healthcare professionals favored WIC screening using the EHR but workflow challenges existed (CFIR: implementation process)

Healthcare professionals were asked about the process of screening patients for WIC enrollment following implementation of WIC EHR access. Most, but not all, healthcare professionals described screening patients for WIC as easy because it was standardized, built into EHR visit templates, and triggered an automated referral. Some healthcare professionals identified screening barriers related to workflow, including that screening did not occur during acute visits and sometimes required navigation between multiple screens. Another barrier addressed by healthcare staff was patients not feeling comfortable disclosing their need or eligibility for WIC or feeling offended by the screening questions. For example, one healthcare professional mentioned rewording the questions due to the potential discomfort. Another healthcare professional also mentioned time as a potential barrier to completing screening.

### Theme 3: EHR connections between WIC and the healthcare system can streamline referrals to and enrollment in WIC (CFIR: innovation)

The integration of electronic referrals to WIC within the EHR system allowed for a more streamlined referral process, which one WIC staff stated has led to an increase in their referral numbers. The enrollment and recertification process has also been somewhat improved with the EHR connection; however, barriers to enrollment still exist. We identified 2 subthemes within this primary theme.

#### Subtheme 3.1: prior to the EHR connection, sending referrals to WIC was burdensome on healthcare professionals (CFIR: outer setting) but improved after the intervention (CFIR: innovation)

##### WIC staff

WIC staff discussed various ways they received WIC referrals from healthcare professionals prior to the intervention – primarily by fax and phone. Some WIC staff said that healthcare professionals would walk clients to the WIC clinic or use paper referral forms. This was unique to one WIC office which was colocated within a healthcare clinic. Before the intervention, the frequency with which WIC staff received WIC referrals from healthcare professionals was much lower. They did not specify the frequency of referrals following EHR access implementation but noted that referrals had “really jumped.” Despite this “jump” in referrals, WIC staff expressed that although the intervention may have slightly increased their workflow, it was a welcome tool to better assist clients.

##### Healthcare professionals

Healthcare professionals said that they would most commonly fax prescription forms to the WIC office prior to the intervention. Other less common referral methods were phone calls, giving patients the WIC phone number or referral form to take to the WIC office, walking patients to the WIC office (colocated within one healthcare clinic), or referring through a patient navigator. Several healthcare professionals discussed challenges referring patients to WIC before WIC had EHR access, including their office forgetting to send the fax, paperwork and faxes getting lost, and not getting confirmation from WIC that patients were successfully enrolled. Following the intervention, healthcare professionals discussed how referring patients to WIC had become easier and more streamlined. They felt that referrals were more likely to be successful and that the time burden on healthcare professionals was lessened.

#### Subtheme 3.2: Prior to the EHR connection, WIC staff felt enrollment and recertification were not difficult (CFIR: outer setting), but access to the EHR enabled obtaining required information that made the process more efficient and accurate (CFIR: innovation), while healthcare professionals felt there were barriers (CFIR: outer setting)

##### WIC staff

WIC staff described the WIC enrollment and recertification (i.e., verification that a WIC participant continues to meet benefit requirements) processes as generally easy prior to having EHR access. Some elaborated that the COVID waivers had mitigated potential barriers for clients because they were able to enroll via phone rather than an in-person appointment, and because height, weight, and hemoglobin requirements had been waived. Several WIC staff described WIC-client communication challenges, including WIC staff having difficulty reaching potential clients by phone and vice versa. Most WIC staff felt that the WIC enrollment and recertification process was easier after the intervention due to having client information (e.g., anthropometric measurements).

##### Healthcare professionals

Healthcare professionals also noted patient difficulty attending in-person visits at the WIC office prior to COVID waivers as a barrier to WIC enrollment for their patients. Other barriers identified included lack of awareness of WIC services or eligibility, shame, or stigma about accepting WIC services, and concerns from undocumented patients that filling out WIC paperwork or receiving benefits would negatively affect them.

### Theme 4: WIC staff and healthcare professionals recommended that WIC have EHR access (CFIR: implementation process)

Most WIC staff and healthcare professionals recommended that WIC have the option of EHR access to improve communication and coordination between the two parties. We identified two subthemes within this primary theme.

#### Subtheme 4.1: WIC staff and healthcare professionals expressed that access to EHR may improve communication and coordination (CFIR: innovation)

##### WIC staff

Following the intervention, all WIC staff accessed client medical records to obtain or confirm anthropometric measurements, and all but one said that they used it to access hemoglobin and/or lead test results. Several said that they accessed the healthcare provider’s notes or other documentation from visits to learn about or to verify a client’s medical history, the name of a condition, and/or to understand the discussion between client and provider. WIC staff enthusiastically recommended that WIC offices have access to client’s EHRs going forward. All WIC staff described benefits of having access to a read-only version of the EHR, including being able to verify information from clients and/or obtain accurate information when clients could not recall exact anthropometric measurements; facilitating phone consultations with clients; and improved communication and feelings of connectedness between WIC staff and healthcare professionals.

##### Healthcare professionals

All pediatricians and the RN recommended that WIC offices have access to patients’ EHRs. They felt that access would facilitate communication and care coordination and that it was beneficial for patients if their WIC provider and healthcare professionals shared information and communicated similar messages. They identified the benefits and efficacy of the intervention as an increased percentage of successful WIC referrals and higher WIC enrollment, and therefore, more patients accessing services. Medical assistants and the nurse were less supportive of WIC’s EHR access and a couple expressed that they did not think having access was necessary to determine whether a patient needed WIC because anthropometric data could be obtained without EHR access.

#### Subtheme 4.2: Direct messaging between WIC and healthcare professionals via the EHR could potentially improve communication and coordination (CFIR: innovation), but many were unaware of the function (CFIR: individuals)

##### WIC staff

Following the intervention, most WIC staff communicated with healthcare professionals via the EHR infrequently. For some, this was because they did not have any communication needs, while others did not specify. When they did send and receive messages, WIC staff described the response time as faster than before having EHR access (e.g., within a day or two, an hour or less, etc.). A few WIC staff also said that they spoke with healthcare professionals less frequently by phone or fax after EHR access was implemented. Most WIC staff said that questions about prescriptions were still the most common reason they would send a message to a healthcare professional.

##### Healthcare professionals

Following the intervention, most healthcare professionals sent referrals through the EHR, and one said that they sent formula prescriptions through the EHR. None had used the EHR to send or receive messages due to a lack of training. Many were not aware they could send messages to WIC or were not sure how or to whom to send messages since they did not know the names of all WIC staff. Others had not encountered a need to send a message. Some healthcare professionals said that they communicated with the WIC office via phone or fax less following WIC EHR access implementation. Another said that they faxed WIC less because they sent formula prescriptions through the EHR but spoke on the phone with WIC the same amount. Others felt that their phone and fax communication with WIC had not changed, because they were not aware of how to use the EHR to send messages.

## Discussion

This qualitative study explored perceptions of a novel EHR intervention to facilitate referrals to and enrollment in WIC and increase communication and care coordination between WIC and our healthcare system. Semi-structured interviews with WIC staff, pediatricians, MAs, and a nurse revealed broad support for the program. Most healthcare professionals recommended that WIC have EHR access. Participants overwhelmingly reported that the intervention increased care coordination and streamlined WIC referrals from healthcare professionals, but lack of knowledge about the ability to send EHR messages between WIC and healthcare professionals limited communication. These results indicate that having an EHR connection is beneficial for WIC staff and healthcare providers alike, including improving communication and care coordination and increasing enrollment and retention in WIC, which have been linked with improved health outcomes.

To our knowledge, this study is one of the few to evaluate perceptions of an innovative EHR-based WIC screening and referral tool and electronic data sharing with WIC. One prior qualitative study from the WIC Enhancements to Early Health Lifestyles for Baby (WEE Baby) intervention involving automated, bi-directional, and continuous data sharing between WIC and healthcare electronic systems demonstrated similar findings that integrated data systems could encourage WIC-healthcare information sharing to improve care coordination, and a quantitative study revealed feasibility and usability of data sharing among all parties [17,18]. Another quantitative WEE Baby study demonstrated that although the intervention did not reduce rapid infant weight gain, it was associated with modestly lower weight for age *z*-scores and body mass index among infants, indicating potential health benefits of EHR-based care coordination between WIC and the healthcare system [16]. Another quality improvement study within our health system showed the feasibility of integrating WIC screening and referral in a primary care setting [20]. These few studies regarding EHR data sharing between WIC and health care systems indicate a need for more EHR-based implementation studies and research regarding data sharing, including WIC screening and referral.

Utilizing the CFIR framework, our study revealed potential challenges to implementation across three domains. For example, within the “individuals” domain, some healthcare professionals described a lack of knowledge around the direct messaging capability between WIC staff and healthcare professionals, limiting communication and care coordination. Although WIC staff received training, some still expressed a lack of knowledge of EHR capability, and healthcare professionals were not trained effectively nor provided names of WIC staff with whom to exchange messages within the EHR. Some of the healthcare professionals, notably the medical assistants, expressed reservations about whether families would feel comfortable answering questions about WIC and were less supportive of WIC having EHR access. The medical assistants may have been more demographically similar to the patient population; these perspectives are worth exploring in future qualitative studies. Within the “implementation process” domain, workflow barriers such as mitigating stigma in the screening process, timing of the automated alert, and additional time needed to screen, refer, or counsel were also described. These challenges will require iterative adaptations to further improve communication and care coordination and to enhance scalability. Within the “outer setting,” several barriers to WIC enrollment were also described, such as difficulty attending in-person appointments, which may require permanent policy solutions. In addition to challenges, several facilitators to implementation were identified across all five CFIR domains, demonstrating that an EHR-based intervention provides an opportunity for cross-sector data sharing and improved care coordination [17,18].

This study could have far-reaching policy implications, such as promoting health equity. Due to long-standing structural racism, discrimination, and xenophobia, minoritized individuals are more likely to experience food insecurity in addition to adverse birth outcomes, which WIC improves [3,5,6,31]. Minoritized individuals face additional challenges in accessing WIC and other government benefit programs, such as discrimination and unfair treatment when applying for benefits [32,33]. Therefore, primary care clinic-integrated referral programs could help address these barriers. From a policy perspective, in 2022 the White House convened a historic conference on hunger, nutrition, and health, during which a national strategy was disseminated, including a goal to “integrate nutrition and health” as one of five pillars of focus. In addition, there was an identified need for health systems-level interventions that could “connect [patients] to resources like SNAP [Supplemental Nutrition Assistance Program], WIC, or local food banks [34].” Therefore, research identifying implementation interventions through which healthcare systems can accomplish this goal has a high likelihood of affecting local, state, and federal policies that reduce health inequities. Additionally, through a cooperative agreement with the United States Department of Agriculture, the Food Research & Action Coalition has funded proposals to “expand partnerships with community organizations and the use of community-level data to develop and test WIC outreach efforts [35],” many of which include WIC-healthcare system interventions. While these interventions are ongoing and have yet to be evaluated, they have the capacity to promote health equity.

### Limitations

There are several limitations to this study that should be acknowledged. First, all participants included were from a single healthcare system that had implemented a novel EHR-based WIC screening and referral intervention, so our results may not be transferable to another institution with different or fewer resources available. Second, several of the WIC staff and healthcare professionals were unaware of specific features within the EHR, including messaging WIC staff, which may have limited the ability to fully explore perceptions of the intervention. Thirdly, participants may not be representative of all WIC staff or healthcare professionals within our system, and our study only included a small subset of each provider role limiting the ability to compare perspectives. Lastly, the client/patient perspective is critical to include with data-sharing interventions, and although not included in this manuscript, it is the focus of ongoing research.

### Implications for practice

Recognizing the potential barriers in data sharing amongst WIC and healthcare systems, there are several opportunities for screening, referral, and care coordination. Working closely with institutional information technology experts and clinical informaticists, WIC enrollment screening, and referral questions can be implemented in the EHR as part of a standard workflow. Alternatively, these screening questions could be paper-based, as has previously been described [20]. Documentation in the EHR could also be standardized to reflect when families consent to a referral to WIC. For consenting families, several options for referral initiation can occur. Some states provide online referral forms, such as North Carolina, that healthcare staff could complete on behalf of the patient [36]. Alternatively, a flyer could be placed in exam rooms encouraging families to self-refer with a quick response code linking to the online referral form. Other states, such as New York, have “WIC Medical Referral Forms” that could be completed by paper and faxed or could be built electronically and faxed as a communication within the EHR [37]. A similar process could occur for WIC prescriptions. As health systems begin to adopt WIC screening and referral programs, to ensure that implementation occurs equitably, tracking of the screening, referral, and enrollment metrics stratified by key demographics will be important to ensure that existing inequities are not exacerbated.

## Conclusion

WIC screening and referral interventions within healthcare settings, and the sharing of EHR data with WIC, have the potential to improve enrollment and retention in WIC, as well as increase communication and care coordination. Several federal initiatives promote opportunities to integrate nutrition and healthcare. However, more research is needed to evaluate the impact of electronic data sharing between WIC and healthcare systems, including on healthcare outcomes.
